# Forward flexion of trunk in Parkinson's disease patients is affected by subjective vertical position

**DOI:** 10.1371/journal.pone.0181210

**Published:** 2017-07-10

**Authors:** Kyohei Mikami, Makoto Shiraishi, Tsubasa Kawasaki, Tsutomu Kamo

**Affiliations:** 1 Department of Rehabilitation, Noborito Neurology Clinic, Kawasaki, Kanagawa, Japan; 2 Department of Neurology, St. Marianna University School of Medicine, Kawasaki, Kanagawa, Japan; 3 Department of Physical Therapy, Faculty of Health Science, Ryotokuji University, Urayasu, Chiba, Japan; 4 Department of Neurology, Noborito Neurology Clinic, Kawasaki, Kanagawa, Japan; Tokai University, JAPAN

## Abstract

**Purpose:**

No method has been established to evaluate the dissociation between subjective and objective vertical positions with respect to the self-awareness of postural deformity in patients with Parkinson’s disease (PD). The purpose of this study was to demonstrate, from the relationship between an assessment of the dissociation of subjective and objective vertical positions of PD patients and an assessment based on established PD clinical evaluation scales, that the dissociation regarding vertical position is a factor in the severity of the forward flexion of trunk (FFT).

**Methods:**

Subjects were 39 PD patients and 15 age-matched healthy individuals (control group). Posture was evaluated with measurement of FFT angle during static standing and the subjective vertical position (SV) of the patient. For evaluation of motor function, the Modified Hoehn & Yahr scale, Unified Parkinson’s Disease Rating Scale (UPDRS), 3-m Timed Up and Go Test (TUG), and Functional Reach Test (FRT) were used.

**Results:**

In PD patients, FFT angle in the 3rd tertile of patients was 13.8±9.7°, significantly greater than those in the control group and the 1st and 2nd tertiles of PD patients (control group vs 3rd tertile, p = 0.008; 1st tertile vs 3rd tertile, p<0.001; 2nd vs 3rd tertile, p = 0.008). In multiple regression analysis for factors in the FFT angle, significant factors were SV, disease duration, and the standard deviation of each SV angle measurement.

**Conclusion:**

The dissociation between SV and objective vertical position affects the FFT of PD patients, suggesting an involvement of non-basal ganglia pathologies.

## Introduction

Postural deformities are a common symptom of Parkinson’s disease (PD). A forward flexion of trunk (FFT) that becomes severe due to progression of the condition is intractable, and no treatment has been established [1,2]. Such a posture also represents a critical factor in decreased activities of daily living [3]. Appropriate perception of one’s own posture is reportedly difficult in PD patients who have developed postural deformity [4,5]. Recently there have been reported the issue that the onset of postural deformities in PD patients might be related to frontal lobe dysfunction as Behavioral Assessment of the Dysexecutive Syndrome score was significantly lower in PD patients with severe postural deformities than without it [6]. On the basis of these reports, factors other than extrapyramidal disorder are conjectured to be involved in the mechanism of onset for postural deformities. Furthermore, some factors (i.e., rigidity [7], drug-induced [8], dystonia [9], proprioceptive disintegration [10]) other than extrapyramidal disorder are conjectured to be involved in the mechanism of onset for postural deformities.

Non-image qualitative evaluation of spine posture using a computer assisted noninvasive device called the spinal mouse in patients with PD might be useful to monitor response to levodopa treatment [11,12]. With regard to the self-awareness of postural deformity, no method has been established to evaluate the dissociation between subjective and objective vertical positions. One of subjective vertical measurements was interpreted to determine vertical position subjectively by tilting the whole body passively [13,14]. The subjective vertical position in this study was defined as the angle of position obtained by gradually tilting not the whole body but the trunk passively. We hypothesized that this dissociation is a factor in the severity of FFT, and that non-basal ganglia pathologies are involved in this condition. The present study aimed to examine the validity of this hypothesis from the relationship between an assessment of the dissociation between subjective and objective vertical positions of PD patients and an assessment based on established PD clinical assessment scales.

## Materials and methods

### Subjects

Subjects were 39 patients who satisfied the following selection criteria and exclusion criteria among PD patients undergoing rehabilitation in our outpatient clinic (17 men, 22 women; mean age, 71.9±10.1 years). Selection criteria were: 1) regular follow-up once a week or once every 2 weeks; 2) provision of consent for Parkinson’s disease assessment performed in routine care; and 3) Stage II to IV on the Modified Hoehn & Yahr scale (H-Y). Exclusion criteria were 1) a score of ≤24 on the Mini Mental State Examination (MMSE); 2) presentation of severe wearing off (≥75% of waking hours); 3) severe psychiatric or autonomic symptoms; 4) sudden postural deterioration (≥10° in one week); 5) difficulty maintaining a standing posture; and 6) trunk extension range of motion ≤5°. A control group comprised 15 healthy elderly individuals (7 men, 8 women; mean age, 68.3±3.3 years) who participated in a health exercise class. No subjects displayed abnormalities in cognitive function, with a score of 6 in the Six-Item Screener Test, a screening test of cognitive function.

Methods and measures used in this study were in accordance with rehabilitation program, and we evaluated all patients undergoing rehabilitation therapy in this clinic. The individual fulfilling criteria applied in this study has given informed consent in writing and orally about this program. These data used for case-control study were approved by the Ethics Committee of the Japan Primary Care Association (approval #, 2015–002).

### Measurement of forward flexion of trunk angle and the vertical position angle felt by patients

**Fig 1:**
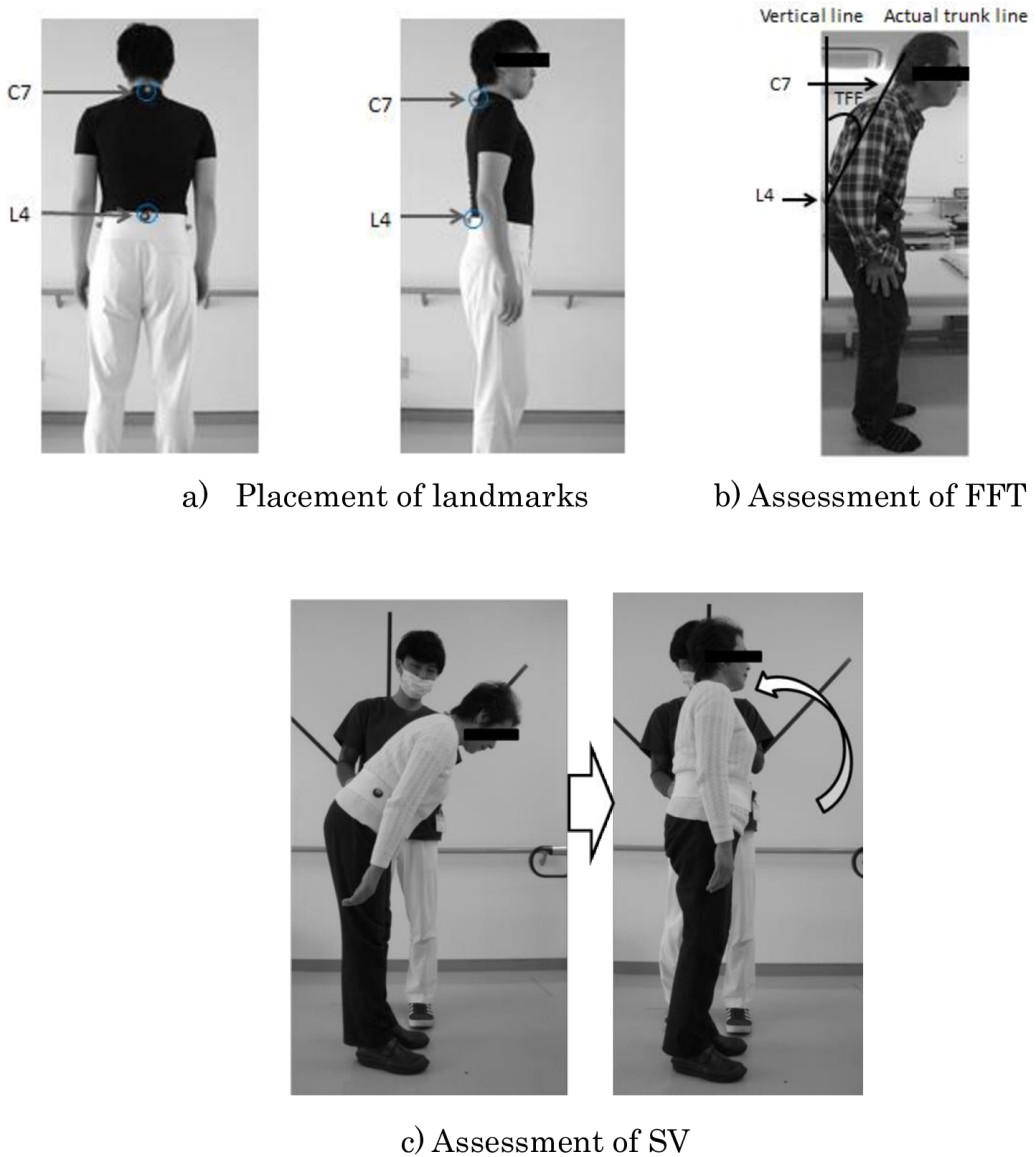
Method of assessing posture. a) Placement of landmarks. b) Assessment of FFT. c) Assessment of SV. C7: 7th cervical vertebra. L4: 4th lumbar vertebra. Vertical line: vertical line to the floor through L4. Actual trunk line: line connecting C7 and L4. FFT: forward flexion of trunk. Angle between the vertical line and actual trunk line. SV: subjective vertical position. Measurement of angle between subjective vertical position and objective vertical position of subjects. The starting position was a closed-eyes standing position in a position of 45° forward flexion. Extension from the 45° forward flexion position to a vertical position was passively induced at a speed of about 5 s, and the subject reported to the examiner the point in time that they recognized as vertical. Measurements were performed three times, and the mean of those three measurements was taken as SV.

Angle of FFT and subjective vertical position (SV) in a static standing position were measured in the PD group and control group. For measurements of FFT and SV, images were taken with markers attached to the 7th cervical vertebra (C7) and 4th lumbar vertebra (L4), and measurements were made using free Image J software (Fig 1A). FFT was measured as the angle between the vertical line and the actual trunk line, with the vertical line defined as the line that passed vertically through L4 to the floor and the actual trunk line defined as the line connecting C7 and L4 (Fig 1B). The subjective vertical position (SV) was measured as the angle between the vertical line and the position recognized by the individual patient as the vertical position (Fig 1C). In measuring SV, subjects maintained a standing position with eyes closed, after which the trunk was passively guided to a forward flexion angle of 45°. Next, the subject was passively moved at a speed of about 5 s from the position with FFT of 45° to 0°, and reported the position perceived as the vertical position. This was performed 3 times, and the mean value of these 3 values was taken as SV. The standard deviation of the 3 measurements was calculated as the variability of 3 measurements. FFT was measured to remain standing voluntarily, while SV was moved trunk passively from the 45° forward flexion position to a vertical position in front of the line illustrated angles on the wall. Vertical line through L4 was measured based on the Measurement of the range of motion in joints by The Japanese association of rehabilitation medicine [15].

Motor function of the PD patient was assessed with H-Y, Unified Parkinson’s Disease Rating Scale (UPDRS) part I to IV, 3-m Timed Up-and-Go test (TUG), and Functional Reach Test (FRT).

### Statistical analyses

Values are shown as mean ± standard deviation. In comparisons between the two groups, continuous variables that did not show a normal distribution were analyzed using the Mann-Whitney U-test. For differences in each factor by FFT tertile, continuous variables were analyzed using analysis of variance and ratio differences were analyzed using Pearson’s *χ*^2^ test. In cases where a significant difference was obtained in comparisons between ≥3 groups, the post-hoc test used to determine which groups showed differences was Dunnett’s test in comparisons with the FFT of the control group as controls, and Bonferroni’s method in 4-group comparisons of SV in the control group and FFT tertiles of the PD group. Relationships with factors affecting FFT were analyzed with multiple regression analysis (step-wise regression model). SPSS version 21 statistical software (IBM SPSS Statistics for Windows; IBM, Armonk, NY) was used, with values of p < 0.05 taken to indicate a significant difference.

## Results

**Fig 2:**
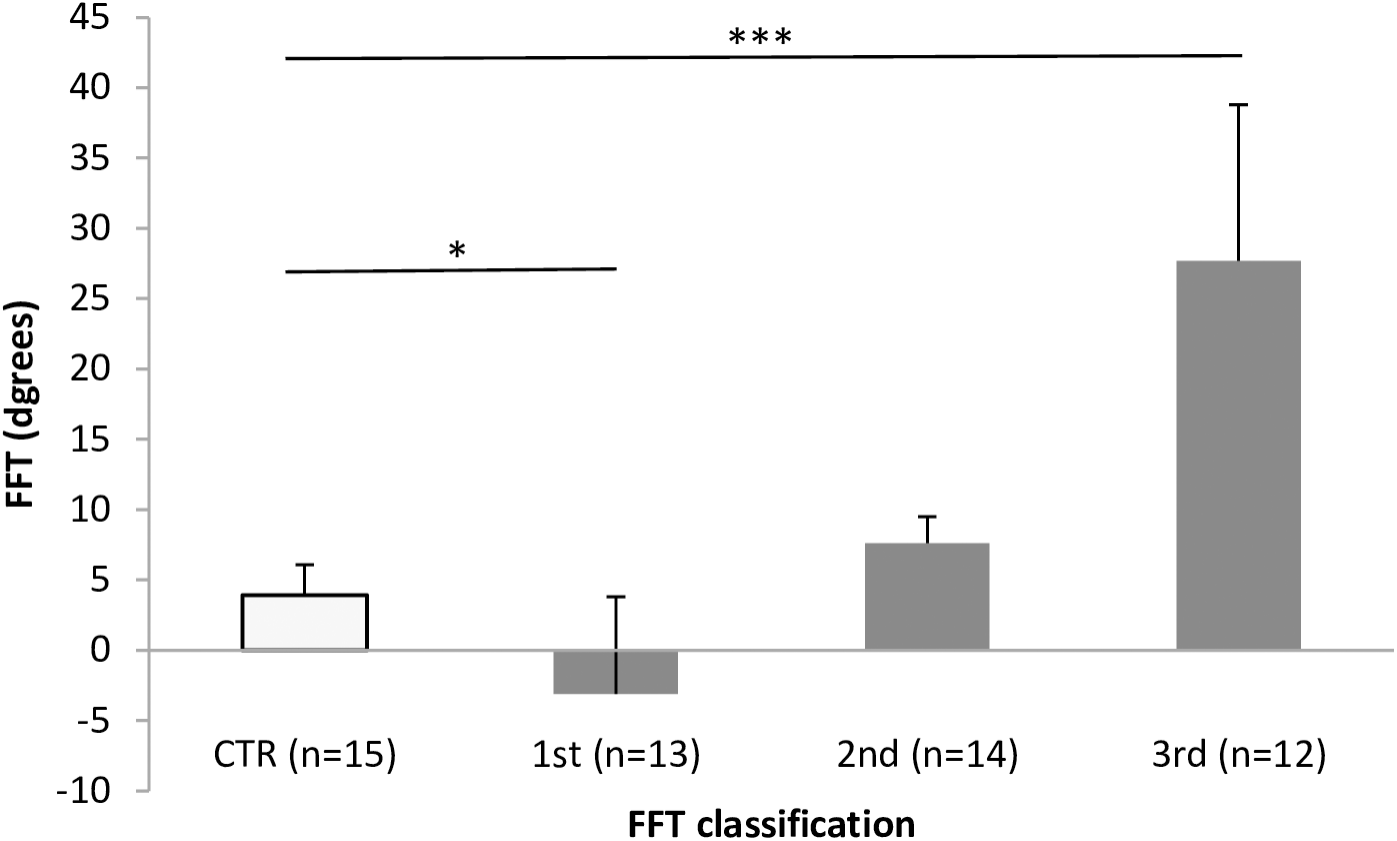
Comparison of FFT angle in control group and FFT tertiles. Significant differences with FFT angle of the control group are apparent in the 1st tertile (p = 0.02) and 3rd tertile (p<0.001). No significant difference is seen in the 2nd tertile (p = 0.36). *p < 0.05; ***p < 0.001. FFT: forward flexion of trunk.

Baseline characteristics for the PD group are shown in Table 1. FFT was 10.2±14.7° in the PD group overall. Compared with the 3.9±2.6° in the 15 control patients (mean age, 68.3±3.3 years; 7 men), TFF was significantly larger in the PD group (p = 0.032). The baseline characteristics of FFT tertiles in the PD group are shown in Table 2. No differences in age, disease duration, MMSE, H-Y, UDPRS parts, TUG, FRT, levodopa dosage, or dopamine agonist dosage rate were seen between groups (Table 2). In contrast, compared with the FFT of 3.9±2.6° in the control group, the 1st FFT tertile (-15° to 4°) was significantly lower at -3.1±7.2° (p = 0.045), and the 3rd tertile (13° to 45°) was significantly higher at 27.7±11.6° (p < 0.001) (Fig 2).

**Table 1:** Baseline characteristics fort the PD group.

	PD(n = 39)
**Age (y)**	71.9 ± 10.1
**Sex (Male: Female)**	17: 22
**Disease duration (y)**	7.2 ± 5.4
**MMSE (points)**	27.8 ± 2.1
**H-Y (stage)**	2.6 ± 0.7
**UPDRS part I (points)**	2.1 ± 1.4
**UPDRS part II (points)**	7.9 ± 5.8
**UPDRS part III (points)**	15.3 ± 9.4
**UPDRS part IV (points)**	2.5 ± 2.7
**UPDRS total (points)**	24.4 ± 13.2
**FFT (degrees)**	10.2 ± 14.7
**SV (degrees)**	5.20 ± 1.1
**TUG (sec)**	12.8 ± 9.4
**FRT (cm)**	23.8 ± 8.2
**L-dopa (mg)**	375.6 ± 114.6
**Dopamine agonist (%)** **Ropinirole** **Rotigotine** **Pramipexole**	94.9 35.9 35.9 25.6

MMSE: Mini Mental State Examination

H-Y: Hoehn and Yahr scale

UPDRS: Unified Parkinson’s Disease Rating Scale

FFT: forward flexion of trunk

SV: subjective vertical position

TUG: 3-m Timed Up and Go test

FRT: Functional Reach Test

**Table 2:** FFT characteristics of tertile.

	forward flexion of trunk (°)			
Tertile	1st (n = 13) <5°	2nd (n = 14) ≧5°,≦10°	3rd (n = 12) >10°	P value
**Age (y)**	70.2±14.1	70.9±7.8	74.8±6.8	0.30
**Disease duration (y)**	4.8 ± 3.2	8.3 ± 7.7	8.6 ± 3.2	0.15
**MMSE (points)**	27.6 ± 2.2	28.6 ± 1.9	26.9 ± 1.9	0.10
**H-Y (stage)**	2.4 ± 0.8	2.5 ± 0.8	2.8 ± 0.6	0.51
**UPDRS Part I**	1.8±1.5	2.4±1.3	2.3±1.3	0.55
**UPDRS Part II**	5.5±4.4	7.7±5.5	10.8±5.8	0.07
**UPDRS Part III**	18.2 ± 12.1	13.7 ± 6.5	20.4 ± 7.9	0.06
**UPDRS Part IV**	1.4±1.9	3.5±2.9	2.3±2.2	0.09
**UPDRS total**	23.8±15.2	24.9±10.9	35.3±12.8	0.08
**TUG (sec)**	9.2 ± 1.4	8.9 ± 3.8	18.5 ± 12.4	0.13
**FRT (cm)**	23.0 ± 8.8	23.3 ± 9.6	22.1 ± 7.8	0.24
**Variability of 3times (°)**	1.7 ± 1.5	1.9 ± 1.8	2.6 ± 1.8	0.43
**L-dopa (mg)**	311.5 ± 93.9	414.3 ± 121.6	400.0 ± 194.2	0.14
**Agonist (%)**	92.3	100	91.7	0.12

FFT: forward flexion of trunk

MMSE: Mini Mental State Examination

H-Y: Hoehn and Yahr scale

UPDRS: Unified Parkinson’s Disease Rating Scale

TUG:3-m Timed U- and-Go test

FRT: Functional Reach Test

**Fig 3:**
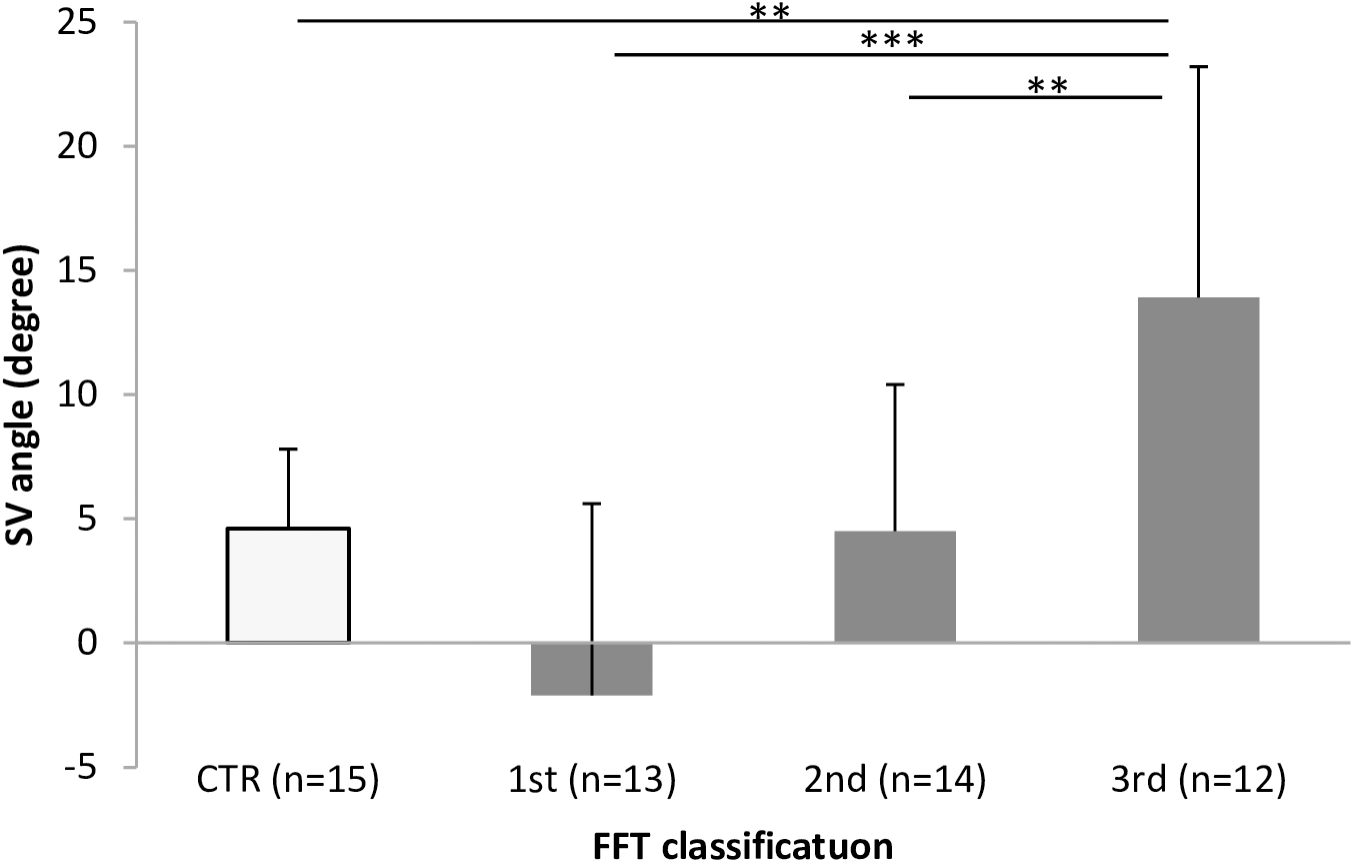
SV of control group and each FFT tertile group. A significant difference in SV was observed between the 3rd tertile and other groups (control group vs. 3rd tertile, p = 0.008; 1st tertile vs. 3rd tertile, p = 0.000; 2nd vs. 3rd tertile, p = 0.008). *p < 0.05; **p < 0.01; ***p < 0.001. SV: subjective vertical position. FFT: forward flexion of trunk.

Reliability from SV measurements was 0.728 in the control group and 0.927 in the PD group. In FFT tertiles, reliability was 0.869 in the 1st tertile, 0.837 in the 2nd tertile, and 0.938 in the 3rd tertile. SV was 5.2±10.1° in the PD group overall and 4.6±3.3° in the control group. SV of the 3rd tertile (FFT 13° to 45°) in the PD group was 13.8±9.7°, significantly higher than the SV of 4.6±3.2° in the control group (FFT 0° to 9°), SV of -2.1±7.7 in the 1st tertile (FFT -13° to 4°), and SV of 4.5±5.9° in the 2nd tertile (FFT 5° to 10°) (control group vs 3rd tertile p = 0.008, 1st tertile vs 3rd tertile p<0.000, 2nd vs 3rd tertile p = 0.008, Fig 3).

Multiple linear regression analyses were performed to model FFT with the following variables: age, sex, disease duration, UPDRS III, SV, and standard deviation of SV (variability of 3 measurements) as independent variables. SV, standard deviation of SV, and disease duration were independently correlated with FFT (Table 3).

**Table 3:** Multiple linear regression analyses for FFT.

	*B*	SE*b*	Beta	p value
**Model 1**				
**SV (°)**	1.009	0.161	0.737	< 0.001
**Model 2**				
**SV**	1.178	0.136	0.812	< 0.001
**Variability of 3 measurements (°)**	3.452	0.818	0.396	< 0.001
**Model 3**				
**SV (°)**	1.143	0.131	0.788	< 0.001
**Variability of 3 measurements (°)**	3.285	0.787	0.377	< 0.001
**Disease duration (y)**	0.499	0.241	0.185	0.046

FFT: forward flexion of trunk

SV: subjective vertical position

## Discussion

The finding that the dissociation between subjective vertical position and objective vertical position in PD patients shown in this study was a factor in the severity of the FFT supported our hypothesis.

In this study, the vertical position felt by PD patients themselves was displaced toward greater forward flexion in contrast to the actual vertical position. This was the first study to investigate the increase in FFT of PD patients from the perspective of SV. The postural deformity of PD patients is known to comprise multiple factors, one of which has been suggested to be proprioceptive disintegration [16]. For example, in an eyes-closed condition on a dynamic platform, PD patients show greater difficulty maintaining a vertical position posture than healthy adults, but this reportedly improves with open eyes [17], and in forward-reach movements performed with the eyes open and closed, significant displacement of the center of mass under the eyes-closed condition compared to the eyes-open condition is not seen in healthy individuals, but occurs in PD patients [18]. This study supports the above investigations.

In this study, disease duration was included in the factors related to FFT (Table 3). Postural abnormalities often occur in the late stage of the disease [1,19]. Similar results were found in this study. In contrast, involvement of H-Y, UPDRS, FRT, and TUG in FFT was not shown. As a factor in this result, deterioration of the FFT was interpreted as being independent of other motor signs. Deterioration of postural deformities reportedly parallels disease progression [20–22], and the rate of motor symptom progression in fact slows as the condition develops or disease duration increases [23], but no consensus of opinion has been reached.

Non-basal ganglia conditions are also reportedly involved in postural deformity [24–27], and in addition to abnormalities in basal ganglia function that originate in dopaminergic neuron degeneration of the midbrain substantia nigra, some kind of non-dopaminergic or non-basal ganglia functional degeneration in PD of long duration is conjectured to cause the vertical position recognized by the aforementioned patients themselves to become poorer and FFT to deteriorate. In a past study on QOL in PD patients, we reported that FFT does not necessarily coincide with feelings of difficulty in movement [3], and stressed that the posture of PD patients differs from the mechanisms of other motor symptoms. The posture assessment used in this study is simple and does not use any special test instruments, and so may be clinically useful. In the future, the effects need to be verified in an interventional trial to examine the question of whether non-basal ganglia conditions are involved in the FFT in PD.

Several limitations need to be considered when interpreting the results of this study. First, the lack of cognitive dysfunction or severe psychiatric symptoms were criteria for inclusion as subjects, and the relationship between postural deformities and non-motor symptoms such as depression or dementia remains unclear. Huber *et al*.[28] reported that although severity of PD was unrelated to depression, a negative correlation was seen with cognitive function. Further investigation is needed on the relationship between postural deformities in PD patients, depression, and dementia. Second, the sample size in this study was small, and significant differences thus may not have been adequately detected. Third, this was a cross-sectional investigation and in order to verify the effects of long-term disease, it is necessary to observe changes over time and to investigate therapeutic interventions.

In conclusion, the FFT in PD patients is affected by a dissociation between subjective and objective vertical positions, suggesting involvement of non-basal ganglia pathologies.
